# ADHD and later-life labor market outcomes in the United States

**DOI:** 10.1007/s10198-019-01055-0

**Published:** 2019-05-02

**Authors:** Cornelius A. Rietveld, Pankaj C. Patel

**Affiliations:** 1 Erasmus School of Economics, Erasmus University Rotterdam, Burgemeester Oudlaan 50, 3061 PA Rotterdam, The Netherlands; 2 Villanova School of Business, Villanova University, 800 E. Lancaster Avenue, Villanova, PA 19085 USA

**Keywords:** ADHD, Educational attainment, Labor market outcomes, Polygenic risk score, I14, J01

## Abstract

This study analyzes the relation between attention-deficit hyperactivity disorder (ADHD) and later-life labor market outcomes in the United States and whether these relationships are mediated by educational attainment. To overcome endogeneity concerns in the estimation of these relationships, we exploit the polygenic risk score (PRS) for ADHD in a cohort where the diagnosis of and treatment for ADHD were generally not available. We find that an increase in the PRS for ADHD reduces the likelihood of employment, individual income, and household wealth. Moreover, it increases the likelihood of receiving social security disability benefits, unemployment or worker compensation, and other governmental transfers. We provide evidence that educational attainment mediates these relationships to a considerable extent (14–58%).

## Introduction

Attention-deficit hyperactivity disorder (ADHD) is a neurobehavioral developmental disorder that is characterized by inattention, hyperactivity (restlessness), disruptive behavior, and impulsivity [17]. A recent meta-analysis estimates the population prevalence of ADHD among children in the range of 5.9–7.1% [23]. ADHD symptoms persist in approximately 60–70% of adults [4, 8, 16]. The estimates of productivity and income losses from ADHD in the US were estimated to be between $87 billion and $138 billion per year, which make ADHD a major public health issue [10].

The impairments in problem solving, planning, and understanding the actions of others have led most ADHD studies to focus on the influence of ADHD on school performance. For example, studies using a sibling fixed-effects model have shown that having ADHD symptoms is negatively associated with test scores and educational attainment [5, 12]. The effect of ADHD on the (youth) labor market outcomes was not known until Fletcher [11] provided evidence in a sample of individuals aged 24–35 that (self-reported) ADHD lowers the likelihood of employment and earnings and increases the likelihood of receiving social assistance. The purpose of the present study is to estimate the effects of ADHD on later-life labor market outcomes.

One of the primary challenges in assessing the relation between ADHD and labor market outcomes is to deal adequately with endogeneity, particularly the measurement error in ADHD and the mutual causality between the manifestation of ADHD symptoms and labor market outcomes. Regarding measurement error, most studies have generally relied on a survey-based dichotomous measure of ADHD diagnoses (yes/no) and the age of ADHD diagnoses [10, 11]. Nevertheless, systematic variations in opportunities for diagnoses available to different cohorts and the filial resources available to cope with ADHD could influence the reporting of ADHD and later-life outcomes.

Studies relying on self-reported ADHD symptoms or diagnoses may also suffer from reverse causality, meaning that labor market experiences may influence the manifestation and reporting of ADHD symptoms. For example, Fletcher [11] draws on retrospective self-reports about whether the respondent was ever told by a doctor, nurse, or other health care provider that the respondent had ADHD. The stratified analysis by Fletcher [11] of those with an early (before age 12) or late ADHD diagnosis (after age 12) shows that those with early diagnosis of ADHD symptoms were driving the results. Within such a design, reverse causality concerns are reduced. However, trailing effects of labor market experiences may still influence the experience of ADHD symptoms. Relatedly, Verheul et al. [22] studies among students how self-reported ADHD symptoms are related to the intention of starting an own business. By drawing on a sample of individuals without experience in the labor market, reverse causality concerns are reduced. However, intentions do not necessarily result in an actual business start-up.

To deal with the above-described endogeneity concerns, we exploit recent advances in unraveling the genetic architecture of ADHD. The heritability of ADHD is in the range of 70–80% [9], meaning that around three-quarters of the differences between individuals in terms of ADHD can be explained by genetic factors. Demontis et al. [6] show that the heritable liability to ADHD is continuously distributed in the population. The clinical status of ADHD is related to a high value on this liability scale. A recent Genome-Wide Association Study (GWAS) succeeded in finding several individual genetic variants that are related to ADHD [6]. Based on the GWAS results, a polygenic risk score (PRS) for ADHD can be constructed. Stergiakouli et al. [20] and Demontis et al. [6] show that this score is a significant predictor of the clinical ADHD status.

This paper investigates the association between ADHD and later-life labor market outcomes using the PRS for ADHD. The PRS for ADHD materializes at conception, and hence we circumvent the measurement issues around the diagnosis of ADHD as well as issues of reverse causality because labor market outcomes cannot change an individual’s value of the PRS for ADHD. Moreover, we draw upon a representative sample of individuals between 50 and 65 years of age (and their spouses) from the Health and Retirement Study, a cohort where the diagnosis of and treatment for ADHD were generally not available. As such, the sample allows for estimations of later-life labor market outcomes that are less biased by time-trends related to diagnoses and treatments of ADHD.

Our approach relates to studies using sibling-fixed effects. However, sibling fixed-effects control for the unmeasured time-invariant genetic and environmental factors. Moreover, sibling fixed-effects do not parse out the relative effects of genes and the environment. With a higher prevalence of ADHD among boys than among girls [23], sibling fixed-effects for boy-girl sibling pairs could bias the estimation of effects. Hence, the use of the PRS for ADHD is instrumental in lowering estimation bias resulting from time-invariant genetic effects.

Our results are generally in line with the study by Fletcher [11] on the relation between ADHD and early-life labor market outcomes (for those between the ages of 24–35). Our results do also suggest a negative relationship between the PRS for ADHD and employment, income and household wealth. Furthermore, the PRS for ADHD is also positively associated with the likelihood of receiving social security disability benefits, receiving unemployment or worker compensation, and receiving other governmental transfers. As a further contribution, we show that PRS for ADHD is negatively associated with the labor market outcomes through lower educational attainment.

## Methods

### Sample

To investigate the relation between ADHD and later-life labor market outcomes, we draw upon longitudinal data from the Health and Retirement Study (HRS). The HRS is an ongoing representative panel of Americans aged 50 and over and their spouses. In this study, we use the PRS for ADHD released in April 2018. This PRS for ADHD is based on the GWAS on ADHD by Demontis et al. [6]. We merged the PRS with the HRS data as provided by the RAND Corporation (Version P, 1992–2014)^1^ [3]. This file contains harmonized data of all available HRS data-collection waves. Since the HRS samples’ individuals aged 50 years or above, we restrict the sample to those aged between 50 and 65 to exclude individuals working beyond the official retirement age in the US. The 50 + restriction is needed, because some of the spouses are younger than 50. Moreover, we restrict the sample to individuals of European ancestry, as recommended by the center responsible for genotyping the HRS participants [21]. Our final sample includes 9033 individuals representing 43,485 individual-year observations with full information on all variables included in the analysis. Table 1 presents descriptive statistics of the analysis sample.

**Table 1:** Descriptive statistics analysis sample

	Females and males *N*_individuals_ = 9033 *N*_individual-wave_ = 43,485		Females *N*_individuals_ = 4921 *N*_individual-wave_ = 24,428		Males *N*_individuals_ = 4112 *N*_individual-wave_ = 19,057	
	Mean	SD	Mean	SD	Mean	SD
*Outcome variables*						
Employed (1 = yes; 0 = no)	0.692	0.462	0.651	0.477	0.746	0.436
Log of earnings	6.790	4.898	6.343	4.852	7.362	4.897
Log of household wealth	12.059	1.774	11.997	1.862	12.140	1.652
Receiving social security disability benefits (1 = yes; 0 = no)	0.045	0.207	0.044	0.204	0.046	0.210
Receiving unemployment/worker compensation (1 = yes; 0 = no)	0.046	0.210	0.035	0.185	0.060	0.237
Receiving other governmental transfers (1 = yes; 0 = no)	0.053	0.223	0.037	0.188	0.073	0.260
*Main independent variable*						
ADHD polygenic score	0.001	1.001	0.014	1.004	− 0.017	0.997
*Mediating variable*						
Years of education (0–17 + years)	13.497	2.418	13.369	2.267	13.661	2.589
*Control variables*						
Age (years)	58.212	4.196	57.985	4.270	58.503	4.082
Gender (0 = male; 1 = female)	0.562	0.496	1.000	0.000	0.000	0.000
With a partner (1 = yes; 0 = no)	0.811	0.391	0.769	0.422	0.866	0.340
Number of living children	2.921	1.816	2.979	1.844	2.847	1.777
Self-reported health (1 = excellent)	0.195	0.396	0.199	0.399	0.191	0.393
Self-reported health (1 = very good)	0.379	0.485	0.385	0.487	0.371	0.483
Self-reported health (1 = good)	0.281	0.450	0.271	0.445	0.294	0.456
Self-reported health (1 = fair)	0.111	0.314	0.111	0.314	0.111	0.314
Self-reported health (1 = poor)	0.034	0.180	0.034	0.182	0.033	0.179
Health limits work (1 = yes; 0 = no)	0.188	0.391	0.200	0.400	0.173	0.378
Tenure in current occupation (years)	17.873	10.268	14.854	9.285	21.744	10.168
Log of spousal earnings	4.999	5.158	4.764	5.246	5.301	5.026

The first ten principal components of the genetic relationship matrix are also included as control variables

*SD* standard deviation

### Empirical setup

In line with previous studies on ADHD and labor market outcomes [11, 15], our primary outcomes are employment (binary indicator whether the respondent is currently working for pay), the logarithm of individual earnings (gross individual income), and the logarithm of total household wealth (net value of total wealth, excluding second home, if applicable). Our secondary outcomes are whether the participant receives governmental assistance in the form of social security disability insurance (binary indicator whether the respondent receives social security disability income), receives unemployment or workers’ compensation (binary indicator whether the respondent receives income from unemployment and worker’s compensation), and receives other governmental transfers (binary indicator whether the respondent receives income from veterans’ benefits, welfare, and food stamps).

Our main explanatory variable is the PRS for ADHD. A PRS is a weighted sum of genetic variants, and the weights are proportional to the estimated effect size of the genetic variant on the outcome of interest in a GWAS [7]. In our case, the weights come from the recent GWAS on ADHD [6]. The score is standardized to have a mean of 0 and standard deviation of 1, to facilitate the interpretation of the effect size estimates. Demontis et al. [6] show that a one standard deviation change in the score is associated with the 26% higher chance of having a clinical ADHD diagnosis. The mediating variable in our study is educational attainment in years of education (0–17 years). Based on the standard practice in genetic studies [18, 19], we include ten principal components of the genetic relationship matrix to control for subtle population stratification. Population stratification may bias associations between genetic factors (such as a PRS) and an outcome if genetic differences between subpopulations in the sample are related to unobserved factors not accounted for in the model. Rietveld et al. [19] have shown that the inclusion of principal components solves this problem adequately in the HRS. Furthermore, we control for the following contemporaneous factors which may be related to labor market outcomes: sex (0 = male, 1 = female), age (years), marital status (1 = with a partner, 0 = without a partner), number of living children, self-reported health (dummies for 1 = excellent to 5 = poor), whether health limits work (1 = yes, 0 = no), tenure in current occupation (years), and the log of spousal earnings.

Consistent with much of the literature examining the associations between health and labor market outcomes, and given the non-time varying measure of the polygenic ADHD score, we use random-effects panel regression. Mediation is assessed using the “difference-in-coefficient” approach [14]. This approach compares the coefficient of the PRS for ADHD in a model with and without the mediating variable. The change in the estimated coefficient for the PRS for ADHD due to the inclusion of the mediating variable indicates to what extent the mediating variable explains the relationship between the PRS for ADHD and the labor market outcomes. The significance of the mediating (indirect) effects is assessed using the method developed by Karlson et al. [13].^2^

## Results

The results in Table 2 show that, in the full sample (Panel A), the PRS for ADHD is significantly associated with all six labor market outcomes in the model without the mediating variable for educational attainment.^3^ We observe that a one standard deviation increase in the PRS for ADHD is associated with a decrease in the likelihood of employment (10.15% lower odds), lower gross individual income (15.80%), and lower household wealth (12.98%). In contrast, an increase in the PRS for ADHD increases the likelihood of receiving social security disability benefits (20.56% higher odds), receiving unemployment or worker compensation (6.72% higher odds), and receiving other governmental transfers (27.38% higher odds). For all outcomes, inclusion of the mediating variable renders the coefficient for the PRS for ADHD closer to zero (Table 3). Together with the significant regression coefficients for educational attainment, this suggests that educational attainment mediates the relation between the PRS for ADHD and the six labor market outcomes considered.

**Table 2:** The relationship between the polygenic risk score (PRS) for ADHD and labor market outcomes (random effects panel regressions)

	(1)	(2)	(3)	(4)	(5)	(6)
	Employed	Log of earnings	Log of household wealth	Receiving social security disability benefits	Receiving unemployment or worker compensation	Receiving other governmental transfers
*Panel A: females and males (N*_*individuals*_ * = 9033, N*_*individual-wave*_ * = 43,485)*						
PRS for ADHD	− 0.107*** (0.037)	− 0.172*** (0.037)	− 0.139*** (0.017)	0.187** (0.081)	0.065* (0.038)	0.242*** (0.066)
*Panel B: females (N*_*individuals*_ * = 4921, N*_*individual-wave*_ * = 24,428)*						
PRS for ADHD	− 0.086* (0.049)	− 0.139*** (0.049)	− 0.158*** (0.024)	0.264** (0.110)	0.083 (0.055)	0.223** (0.093)
*Panel C: males (N*_*individuals*_ * = 4112, N*_*individual-wave*_ * = 19,057)*						
PRS for ADHD	− 0.117** (0.054)	− 0.196*** (0.054)	− 0.118*** (0.024)	0.084 (0.119)	0.057 (0.053)	0.232** (0.102)
*Panel D: females and males aged 50–59 (N*_*individuals*_ * = 8056, N*_*individual-wave*_ * = 25,556)*						
PRS for ADHD	− 0.084* (0.046)	− 0.163*** (0.040)	− 0.128*** (0.019)	0.171 (0.105)	0.093** (0.046)	0.310*** (0.087)
*Panel E: females and males aged 50–55 (N*_*individuals*_ * = 6279, N*_*individual-wave*_ * = 12,907)*						
PRS for ADHD	− 0.090 (0.059)	− 0.157*** (0.047)	− 0.139*** (0.022)	0.049 (0.153)	0.063 (0.064)	0.305*** (0.107)

Full regression results are available in the “Appendix” (Tables 5, 6, 7, 8 and 9)

Standard errors in parentheses

***p < 0.01, **p < 0.05, *p < 0.10

**Table 3:** The relationship between the polygenic risk score (PRS) for ADHD and years of education with labor market outcomes (random effects panel regressions)

	(1)	(2)	(3)	(4)	(5)	(6)
	Employed	Log of earnings	Log of household wealth	Receiving social security disability benefits	Receiving unemployment or worker compensation	Receiving other governmental transfers
*Panel A: females and males (N*_*individuals*_ * = 9033, N*_*individual-wave*_ * = 43,485)*						
PRS for ADHD	− 0.072* (0.037)	− 0.118*** (0.037)	− 0.076*** (0.016)	0.137* (0.082)	0.027 (0.038)	0.204*** (0.067)
Years of education	0.128*** (0.015)	0.196*** (0.015)	0.210*** (0.007)	− 0.212*** (0.033)	− 0.155*** (0.016)	− 0.139*** (0.027)
*Panel B: females (N*_*individuals*_ * = 4921, N*_*individual-wave*_ * = 24,428)*						
PRS for ADHD	− 0.057 (0.049)	− 0.089* (0.049)	− 0.097*** (0.023)	0.212* (0.112)	0.056 (0.055)	0.169* (0.094)
Years of education	0.123*** (0.022)	0.207*** (0.022)	0.232*** (0.010)	− 0.228*** (0.050)	− 0.129*** (0.025)	− 0.235*** (0.044)
*Panel C: males (N*_*individuals*_ * = 4112, N*_*individual-wave*_ * = 19,057)*						
PRS for ADHD	− 0.082 (0.055)	− 0.146*** (0.054)	− 0.052** (0.023)	0.035 (0.121)	0.010 (0.053)	0.256** (0.104)
Years of education	0.114*** (0.021)	0.162*** (0.021)	0.191*** (0.009)	− 0.203*** (0.044)	− 0.177*** (0.021)	− 0.023 (0.039)
*Panel D: females and males aged 50–59 (N*_*individuals*_ * = 8056, N*_*individual-wave*_ * = 25,556)*						
PRS for ADHD	− 0.052 (0.047)	− 0.111*** (0.040)	− 0.069*** (0.018)	0.126 (0.106)	0.048 (0.046)	0.279*** (0.088)
Years of education	0.121*** (0.020)	0.197*** (0.017)	0.207*** (0.008)	− 0.179*** (0.044)	− 0.178*** (0.020)	− 0.135*** (0.036)
*Panel E: females and males aged 50–55 (N*_*individuals*_ * = 6279, N*_*individual-wave*_ * = 12,907)*						
PRS for ADHD	− 0.054 (0.059)	− 0.103** (0.047)	− 0.080*** (0.021)	− 0.020 (0.155)	0.012 (0.064)	0.264** (0.108)
Years of education	0.147*** (0.026)	0.208*** (0.020)	0.213*** (0.009)	− 0.242*** (0.066)	− 0.200*** (0.028)	− 0.182*** (0.045)

Full regression results are available in the “Appendix” (Tables 10, 11, 12, 13, 14)

Standard errors in parentheses

***p < 0.01, **p < 0.05, *p < 0.10

Table 4 (Panel A) provides the estimates of the indirect effect of educational attainment in the relation between the PRS for ADHD and the labor market outcomes in the full sample. The indirect effects equal the effect of the PRS for ADHD on educational attainment multiplied by the effect of educational attainment on the labor market outcome (with some rescaling due to non-linearity in the models with binary outcomes). All six indirect effects are significant (*p*-values < 0.001) and meaningful in terms of effect size because the percentage of the relationship between the PRS for ADHD and labor market outcomes mediated by educational attainment (the indirect effect as percentage of the direct effect of the PRS for ADHD on the outcomes) ranges from 13.92% (receiving other governmental transfers) to 57.62% (receiving unemployment or worker compensation).^4^

**Table 4:** The indirect relationship between the polygenic risk score (PRS) for ADHD and labor market outcomes through educational attainment

	(1)	(2)	(3)	(4)	(5)	(6)
	Employed	Log of earnings	Log of household wealth	Receiving social security disability benefits	Receiving unemployment or worker compensation	Receiving other governmental transfers
*Panel A: females and males (N*_*individuals*_ * = 9033, N*_*individual-wave*_ * = 43,485)*						
Indirect effect via years of education	− 0.031*** (0.004)	− 0.047*** (0.004)	− 0.050*** (0.003)	0.051*** (0.008)	0.037*** (0.004)	0.033*** (0.007)
Proportion of mediation	29.85%	28.46%	39.80%	27.02%	57.62%	13.92%
*Panel B: females (N*_*individuals*_ * = 4921, N*_*individual-wave*_ * = 24,428)*						
Indirect effect via years of education	− 0.026*** (0.005)	− 0.044*** (0.005)	− 0.049*** (0.004)	0.048*** (0.011)	0.027*** (0.006)	0.050*** (0.010)
Proportion of mediation	31.37%	32.83%	33.54%	18.56%	32.75%	22.79%
*Panel C: males (N*_*individuals*_ * = 4112, N*_*individual-wave*_ * = 19,057)*						
Indirect effect via years of education	− 0.031*** (0.006)	− 0.044*** (0.006)	− 0.052*** (0.004)	0.055*** (0.012)	0.048*** (0.006)	0.006 (0.011)
Proportion of mediation	27.47%	23.24%	49.89%	61.19%	83.02%	2.84%
*Panel D: females and males aged 50–59 (N*_*individuals*_ * = 8056, N*_*individual-wave*_ * = 25,556)*						
Indirect effect via years of education	− 0.029*** (0.005)	− 0.047*** (0.005)	− 0.050*** (0.003)	0.043*** (0.011)	0.043*** (0.005)	0.032*** (0.009)
Proportion of mediation	35.80%	29.78%	41.73%	25.43%	47.26%	10.42%
*Panel E: females and males aged 50–55 (N*_*individuals*_ * = 6279, N*_*individual-wave*_ * = 12,907)*						
Indirect effect via years of education	− 0.035*** (0.007)	− 0.049*** (0.006)	− 0.050*** (0.005)	0.057*** (0.016)	0.047*** (0.008)	0.043*** (0.011)
Proportion of mediation	38.93%	32.35%	38.72%	154.67%	79.43%	14.02%

Standard errors in parentheses

***p < 0.01; **p < 0.05; *p < 0.10

We performed additional analyses to assess the robustness of our findings. First, given the higher prevalence of ADHD among males compared to females [23], there is a concern that the main results are driven by sex-based differences in the labor market outcomes (Table 1). Therefore, we repeated the analyses in sex-stratified subsamples. The direct effect estimates are available in Table 2 (panels B and C), and the indirect effects’ estimates are available in Table 4 (Panels B and C). We observe that the direct effects of the PRS for ADHD on the labor market outcomes are very similar in size across sexes. However, the coefficient for the PRS for ADHD is not significant in the model explaining receiving social security disability benefits for males (Table 2, column 4) and in the model explaining receiving unemployment or worker compensation for both females and males (Table 2, column 5). The indirect effect size estimates are also very similar in size and significance between males and females, with the results for receiving other governmental transfers as the exception (Table 4, column 6). The latter indirect effect is not significant among males, primarily because there is no significant relationship between educational attainment and receiving income from veterans’ benefits, welfare, and food stamps (Table 3, column 6). The difference with the significant result among females may be due to the small but positive relationship between educational attainment and veteran status among males.

Second, although its sampling strategy (individuals aged 50 + and their spouses) makes the HRS an appropriate data set to study later-life labor market outcomes, labor-market decisions at these ages are also intertwined with the decision of when to retire. Therefore, we repeated the analyses in (i) the subsample of individual-wave observations with age below 60, and (ii) the subsample of individual-wave observations with age between 50 and 55. For individuals in these age categories, we expect the decision to retire to be less of a confounding factor in our analyses. The direct effect estimates are available in Table 2 (panels D and E), and the indirect effects estimates are available in Table 4 (Panel D and E). The direct and indirect effects are similar in direction and magnitude compared to the main results, although some direct effects are insignificant due to the reduction in sample size. Hence, our main results seem not to be conflated by retirement decisions.

## Discussion and conclusion

The present study contributes to the emerging stream of literature showing the value of using genetic information to understand the determinants of later-life labor market outcomes [1, 2]. We find evidence that the PRS for ADHD is negatively associated with educational attainment, the odds for employment, income, and earnings, and it is positively associated with receiving social security disability benefits, receiving unemployment or worker compensation, and receiving other governmental transfers. The direction of these associations is similar as in the study by Fletcher [11] among young adults. Mediation analyses further show that for our six outcomes, educational attainment is an important mediating channel explaining 14–58% of the association between the PRS for ADHD and labor market outcomes. These effects are very similar in size among males and females.

The present study contributes to an emerging stream of studies incorporating genetic information in micro-economic models [1]. We note two important limitations of our study. First of all, although using the PRS for ADHD helps to overcome reverse causality and measurement issues (as discussed in the introduction), it, however, introduces a secondary type of measurement error. That is, the PRS for ADHD captures the genetic component of ADHD only, while the manifestation of ADHD is also partially dependent on environmental circumstances. Relatedly, the interaction of genetic and environmental factors could drive the intensity of ADHD symptoms. Second, as in other studies using a PRS as a predictor of later life outcomes, the explanatory power of PRS score is relatively small. In developing an understanding of practical effect sizes of PRS scores on life outcomes, its relatively low explanatory power must be considered in making inferences.

Nevertheless, the present study contributes to the literature by highlighting the negative effect of ADHD on labor market outcomes among individuals for whom treatment for ADHD was generally not available, and the considerable mediating effect through educational attainment in this relationship. These results raise the question of whether it may be worthwhile to genetically screen for ADHD at a very young age. It is one of the promises of “genoeconomics” to identify possibilities for targeted interventions by giving genetic information about children to parents to create a developmental environment that is most likely to cultivate the children’s abilities [1]. Testing for one’s genetic predisposition for ADHD at a young age may help to plan interventions to improve educational outcomes of those with higher values for the PRS of ADHD. Early stage interventions may help improve the accumulation of human capital and subsequently later-life labor market outcomes. Hence, the negative link between ADHD and educational attainment may possibly be ameliorated because the PRS of ADHD can be measured years before one can formally diagnose ADHD and start with possible treatments.

However, these benefits must be weighted against the disadvantages of genetic screening. First, before one should start with using the PRS for ADHD as a screening instrument, further research on what exactly makes those with a high genetic propensity for ADHD have relatively low educational attainment is needed. Second, the manifestation of ADHD is not solely determined by genes. Hence, a diagnosis of ADHD based on genes only may result in misclassification. Another possible consequence may be that either private insurers would not insure such individuals, thereby increasing burden on the government to cover such costs. Alternatively, those with a genetic predisposition for ADHD may purchase unemployment insurance, which also may not be insured as someone’s genetic make-up is not the result of random or qausi-random environmental circumstances beyond someone’s control. As such, the burden on governmental programs may increase due the non-insurability of labor market outcomes of individuals with a higher genetic predisposition for ADHD. Clearly, careful ethical consideration of the desirability of genetic screening in the context of ADHD is utmost needed.

## Appendix

See Tables 5, 6, 7, 8, 9, 10, 11, 12, 13, 14, 15 and 16.

**Table 5:** The relationship between the polygenic risk score (PRS) for ADHD and labor market outcomes (random effects panel regressions)

	(1)	(2)	(3)	(4)	(5)	(6)
	Employed	Log of earnings	Log of household wealth	Receiving social security disability benefits	Receiving unemployment or worker compensation	Receiving other governmental transfers
*Panel A: females and males (N*_*individuals*_ * = 9033, N*_*individual-wave*_ * = 43,485)*						
PRS for ADHD	− 0.107*** (0.037)	− 0.172*** (0.037)	− 0.139*** (0.017)	0.187** (0.081)	0.065* (0.038)	0.242*** (0.066)
Age	− 0.297*** (0.006)	− 0.274*** (0.005)	0.046*** (0.001)	0.158*** (0.014)	− 0.075*** (0.007)	0.110*** (0.011)
Female	− 0.985*** (0.076)	− 0.708*** (0.076)	0.160*** (0.034)	− 0.886*** (0.167)	− 0.865*** (0.079)	− 1.703*** (0.141)
With a partner	− 0.880*** (0.077)	− 1.320*** (0.075)	0.809*** (0.023)	− 0.751*** (0.159)	− 0.473*** (0.096)	− 1.108*** (0.131)
Number of living children	− 0.013 (0.018)	0.014 (0.018)	− 0.063*** (0.007)	0.032 (0.038)	0.051*** (0.020)	0.176*** (0.030)
Self-reported health (excellent)	Reference	Reference	Reference	Reference	Reference	Reference
Self-reported health (very good)	− 0.032 (0.060)	− 0.004 (0.056)	− 0.024 (0.015)	0.236 (0.283)	0.184** (0.089)	0.357** (0.149)
Self-reported health (good)	− 0.041 (0.069)	− 0.030 (0.065)	− 0.108*** (0.018)	1.153*** (0.279)	0.365*** (0.095)	0.863*** (0.157)
Self-reported health (fair)	− 0.428*** (0.088)	− 0.274*** (0.085)	− 0.233*** (0.024)	2.169*** (0.286)	0.550*** (0.118)	1.402*** (0.179)
Self-reported health (poor)	− 1.770*** (0.140)	− 1.222*** (0.132)	− 0.470*** (0.037)	2.627*** (0.303)	0.695*** (0.171)	1.777*** (0.224)
Health limits work	0.012*** (0.004)	0.056*** (0.004)	0.031*** (0.001)	− 0.079*** (0.009)	− 0.017*** (0.004)	− 0.057*** (0.007)
Tenure in current occupation	− 2.332*** (0.062)	− 2.097*** (0.060)	− 0.161*** (0.016)	4.892*** (0.184)	0.210*** (0.081)	0.952*** (0.110)
Log of spousal earnings	0.070*** (0.005)	0.139*** (0.005)	0.002 (0.001)	− 0.075*** (0.014)	0.015** (0.007)	− 0.092*** (0.012)
Principal component 1	10.526** (4.142)	7.803* (4.084)	5.938*** (1.879)	8.297 (9.420)	− 6.030 (4.251)	8.289 (7.711)
Principal component 2	1.762 (3.918)	− 0.030 (3.972)	− 11.025*** (1.832)	16.507* (9.427)	− 13.397*** (3.863)	31.420*** (8.235)
Principal component 3	− 1.720 (3.952)	− 0.633 (3.985)	− 2.607 (1.832)	− 13.279 (8.529)	− 1.598 (4.135)	− 7.357 (7.063)
Principal component 4	8.601** (3.928)	− 0.053 (3.972)	− 0.538 (1.829)	1.059 (9.185)	− 6.005 (4.125)	− 5.350 (7.185)
Principal component 5	− 8.848** (4.155)	− 6.337 (4.085)	− 14.545*** (1.879)	4.582 (9.487)	− 1.227 (4.257)	12.920* (7.733)
Principal component 6	− 6.111 (3.853)	− 4.561 (3.890)	− 0.662 (1.792)	− 2.126 (8.351)	− 1.440 (4.033)	2.092 (6.917)
Principal component 7	− 2.904 (3.908)	4.883 (3.953)	0.262 (1.819)	− 1.431 (8.552)	2.079 (4.075)	− 5.509 (7.060)
Principal component 8	− 1.555 (3.850)	0.286 (3.890)	2.533 (1.790)	− 2.326 (8.272)	− 3.334 (4.035)	− 2.536 (6.869)
Principal component 9	− 0.949 (3.868)	4.433 (3.898)	− 1.451 (1.794)	1.037 (8.389)	− 3.674 (4.050)	12.259* (6.925)
Principal component 10	− 4.955 (3.912)	− 9.294** (3.955)	2.823 (1.819)	6.156 (8.479)	− 2.947 (4.097)	14.944** (7.088)
Constant	20.401*** (0.368)	22.964*** (0.278)	8.279*** (0.078)	− 17.253*** (0.948)	0.618 (0.414)	− 12.403*** (0.648)

Full regression results for Table 2 (Panel A)

Standard errors in parentheses

****p *< 0.01, ***p *< 0.05, **p *< 0.10

**Table 6:** The relationship between the polygenic risk score (PRS) for ADHD and labor market outcomes (random effects panel regressions)

	(1)	(2)	(3)	(4)	(5)	(6)
	Employed	Log of earnings	Log of household wealth	Receiving social security disability benefits	Receiving unemployment or worker compensation	Receiving other governmental transfers
*Panel B: females (N*_*individuals*_ * = 4921, N*_*individual-wave*_ * = 24,428)*						
PRS for ADHD	− 0.086* (0.049)	− 0.139*** (0.049)	− 0.158*** (0.024)	0.264** (0.110)	0.083 (0.055)	0.223** (0.093)
Age	− 0.273*** (0.007)	− 0.261*** (0.006)	0.043*** (0.002)	0.160*** (0.019)	− 0.081*** (0.010)	0.025* (0.014)
Female						
With a partner	− 1.210*** (0.094)	− 1.616*** (0.091)	0.888*** (0.030)	− 0.876*** (0.199)	− 0.609*** (0.129)	− 1.885*** (0.179)
Number of living children	− 0.047** (0.024)	− 0.027 (0.024)	− 0.045*** (0.009)	0.078 (0.049)	0.054* (0.028)	0.184*** (0.041)
Self-reported health (excellent)	Reference	Reference	Reference	Reference	Reference	Reference
Self-reported health (very good)	0.045 (0.076)	0.059 (0.072)	− 0.058*** (0.021)	0.546 (0.394)	0.291** (0.133)	0.667*** (0.229)
Self-reported health (good)	0.093 (0.089)	0.076 (0.084)	− 0.151*** (0.025)	1.570*** (0.395)	0.425*** (0.144)	1.181*** (0.243)
Self-reported health (fair)	− 0.301*** (0.115)	− 0.221** (0.111)	− 0.269*** (0.034)	2.456*** (0.405)	0.801*** (0.174)	1.879*** (0.266)
Self-reported health (poor)	− 1.577*** (0.185)	− 0.946*** (0.170)	− 0.479*** (0.051)	2.790*** (0.428)	0.513* (0.269)	2.512*** (0.315)
Health limits work	0.037*** (0.005)	0.093*** (0.005)	0.033*** (0.002)	− 0.065*** (0.013)	− 0.003 (0.006)	− 0.063*** (0.011)
Tenure in current occupation	− 2.306*** (0.082)	− 1.928*** (0.076)	− 0.181*** (0.023)	4.539*** (0.243)	0.164 (0.119)	1.037*** (0.152)
Log of spousal earnings	0.066*** (0.006)	0.133*** (0.006)	0.001 (0.002)	− 0.081*** (0.018)	0.007 (0.011)	− 0.123*** (0.018)
Principal component 1	8.022 (5.600)	7.025 (5.643)	4.710* (2.733)	10.055 (12.748)	− 0.408 (6.101)	10.743 (10.980)
Principal component 2	7.043 (5.266)	− 2.447 (5.377)	− 16.472*** (2.606)	26.909** (13.526)	− 20.985*** (5.450)	27.520** (11.837)
Principal component 3	− 1.673 (5.245)	1.010 (5.330)	− 5.666** (2.575)	− 9.212 (11.483)	− 5.601 (5.940)	− 0.465 (9.729)
Principal component 4	4.783 (5.272)	0.656 (5.355)	− 1.240 (2.592)	5.835 (12.721)	− 5.278 (5.991)	− 6.755 (10.465)
Principal component 5	− 1.470 (5.648)	− 4.729 (5.691)	− 16.330*** (2.752)	− 5.070 (12.847)	− 6.641 (6.186)	3.143 (11.026)
Principal component 6	− 6.629 (5.168)	− 10.116* (5.244)	− 0.901 (2.539)	− 5.149 (11.564)	− 3.995 (5.820)	6.454 (9.863)
Principal component 7	− 0.784 (5.250)	7.570 (5.334)	− 1.951 (2.579)	0.880 (11.739)	− 1.346 (5.952)	− 7.104 (10.096)
Principal component 8	1.503 (5.110)	5.368 (5.190)	0.592 (2.509)	− 5.840 (11.393)	0.291 (5.803)	− 11.807 (9.699)
Principal component 9	− 2.278 (5.214)	− 3.392 (5.286)	− 4.468* (2.557)	13.670 (11.716)	− 9.502 (5.964)	13.640 (9.865)
Principal component 10	− 7.084 (5.227)	− 13.265** (5.317)	6.963*** (2.570)	1.065 (11.666)	− 7.256 (5.867)	10.842 (9.946)
Constant	17.951*** (0.444)	21.228*** (0.353)	8.519*** (0.106)	− 18.692*** (1.309)	− 0.011 (0.606)	− 7.355*** (0.923)

Full regression results for Table 2 (Panel B)

Standard errors in parentheses

****p *< 0.01, ***p *< 0.05, **p *< 0.10

**Table 7:** The relationship between the polygenic risk score (PRS) for ADHD and labor market outcomes (random effects panel regressions)

	(1)	(2)	(3)	(4)	(5)	(6)
	Employed	Log of earnings	Log of household wealth	Receiving social security disability benefits	Receiving unemployment or worker compensation	Receiving other governmental transfers
*Panel C: males (N*_*individuals*_ * = 4112, N*_*individual-wave*_ * = 19,057)*						
PRS for ADHD	− 0.117** (0.054)	− 0.196*** (0.054)	− 0.118*** (0.024)	0.084 (0.119)	0.057 (0.053)	0.232** (0.102)
Age	− 0.344*** (0.010)	− 0.298*** (0.007)	0.051*** (0.002)	0.153*** (0.021)	− 0.071*** (0.010)	0.185*** (0.017)
Female						
With a partner	0.007 (0.132)	− 0.554*** (0.129)	0.660*** (0.036)	− 0.439 (0.272)	− 0.254* (0.145)	0.222 (0.238)
Number of living children	0.048* (0.028)	0.086*** (0.027)	− 0.080*** (0.009)	− 0.037 (0.059)	0.052* (0.028)	0.079 (0.049)
Self-reported health (excellent)	Reference	Reference	Reference	Reference	Reference	Reference
Self-reported health (very good)	− 0.153 (0.099)	− 0.080 (0.087)	0.015 (0.021)	− 0.098 (0.411)	0.091 (0.121)	0.178 (0.209)
Self-reported health (good)	− 0.243** (0.109)	− 0.164* (0.099)	− 0.056** (0.025)	0.706* (0.398)	0.319** (0.128)	0.655*** (0.223)
Self-reported health (fair)	− 0.615*** (0.136)	− 0.336** (0.131)	− 0.192*** (0.033)	1.889*** (0.408)	0.333** (0.161)	1.032*** (0.263)
Self-reported health (poor)	− 2.105*** (0.215)	− 1.624*** (0.205)	− 0.462*** (0.052)	2.501*** (0.432)	0.811*** (0.224)	1.000*** (0.340)
Health limits work	− 0.018*** (0.005)	0.016*** (0.005)	0.029*** (0.002)	− 0.094*** (0.012)	− 0.028*** (0.005)	− 0.055*** (0.010)
Tenure in current occupation	− 2.381*** (0.094)	− 2.328*** (0.094)	− 0.134*** (0.023)	5.340*** (0.289)	0.257** (0.111)	0.833*** (0.166)
Log of spousal earnings	0.079*** (0.008)	0.151*** (0.008)	0.003 (0.002)	− 0.066*** (0.021)	0.023** (0.010)	− 0.077*** (0.016)
Principal component 1	14.428** (6.054)	7.933 (5.774)	7.718*** (2.539)	6.269 (14.434)	− 10.960* (5.928)	5.463 (11.556)
Principal component 2	− 7.591 (5.723)	0.314 (5.740)	− 5.050** (2.534)	2.991 (13.124)	− 6.299 (5.456)	26.450** (12.134)
Principal component 3	1.273 (5.855)	− 0.211 (5.844)	0.726 (2.568)	− 19.526 (12.927)	2.489 (5.748)	− 11.110 (10.990)
Principal component 4	11.809** (5.738)	− 1.775 (5.772)	1.225 (2.541)	− 4.186 (13.441)	− 6.285 (5.685)	− 4.735 (10.910)
Principal component 5	− 19.235*** (6.044)	− 8.837 (5.726)	− 12.476*** (2.522)	13.864 (14.572)	3.253 (5.876)	14.141 (11.529)
Principal component 6	− 5.107 (5.624)	2.201 (5.649)	− 0.477 (2.488)	0.654 (12.162)	1.108 (5.577)	− 0.932 (10.540)
Principal component 7	− 6.071 (5.691)	1.822 (5.726)	3.144 (2.522)	− 3.799 (12.661)	5.564 (5.576)	− 4.082 (10.714)
Principal component 8	− 5.775 (5.698)	− 5.748 (5.719)	4.920* (2.520)	1.687 (12.149)	− 6.705 (5.598)	4.985 (10.538)
Principal component 9	− 1.158 (5.622)	10.997* (5.629)	1.934 (2.478)	− 14.535 (12.197)	1.162 (5.515)	9.875 (10.530)
Principal component 10	− 1.211 (5.749)	− 2.945 (5.764)	− 1.762 (2.536)	11.250 (12.469)	2.448 (5.717)	15.832 (10.887)
Constant	23.066*** (0.623)	24.414*** (0.428)	8.162*** (0.106)	− 16.626*** (1.389)	0.438 (0.559)	− 19.214*** (1.022)

Full regression results for Table 2 (Panel C)

Standard errors in parentheses

****p *< 0.01, ***p *< 0.05, **p *< 0.10

**Table 8:** The relationship between the polygenic risk score (PRS) for ADHD and labor market outcomes (random effects panel regressions)

	(1)	(2)	(3)	(4)	(5)	(6)
	Employed	Log of earnings	Log of household wealth	Receiving social security disability benefits	Receiving unemployment or worker compensation	Receiving other governmental transfers
*Panel D: females and males aged 50–59 (N*_*individuals*_ * = 8056, N*_*individual-wave*_ * = 25,556)*						
PRS for ADHD	− 0.084* (0.046)	− 0.163*** (0.040)	− 0.128*** (0.019)	0.171 (0.105)	0.093** (0.046)	0.310*** (0.087)
Age	− 0.200*** (0.011)	− 0.187*** (0.008)	0.052*** (0.002)	0.177*** (0.029)	− 0.038*** (0.013)	0.074*** (0.022)
Female	− 1.105*** (0.098)	− 0.770*** (0.082)	0.194*** (0.038)	− 1.127*** (0.217)	− 0.878*** (0.094)	− 1.754*** (0.196)
With a partner	− 0.876*** (0.115)	− 1.202*** (0.094)	0.925*** (0.033)	− 0.977*** (0.236)	− 0.388*** (0.124)	− 1.135*** (0.186)
Number of living children	− 0.008 (0.024)	− 0.002 (0.021)	− 0.078*** (0.008)	− 0.008 (0.051)	0.049** (0.024)	0.200*** (0.041)
Self-reported health (excellent)	Reference	Reference	Reference	Reference	Reference	Reference
Self-reported health (very good)	− 0.103 (0.088)	− 0.029 (0.065)	− 0.052*** (0.020)	0.078 (0.391)	0.277** (0.109)	0.322 (0.205)
Self-reported health (good)	− 0.126 (0.099)	− 0.054 (0.076)	− 0.156*** (0.024)	1.020*** (0.377)	0.406*** (0.117)	0.881*** (0.219)
Self-reported health (fair)	− 0.716*** (0.125)	− 0.509*** (0.102)	− 0.340*** (0.032)	2.152*** (0.389)	0.477*** (0.149)	1.424*** (0.249)
Self-reported health (poor)	− 2.313*** (0.190)	− 1.748*** (0.157)	− 0.625*** (0.049)	2.833*** (0.408)	0.769*** (0.210)	1.822*** (0.300)
Health limits work	0.038*** (0.005)	0.066*** (0.004)	0.031*** (0.002)	− 0.087*** (0.012)	− 0.020*** (0.005)	− 0.063*** (0.010)
Tenure in current occupation	− 2.826*** (0.093)	− 2.018*** (0.076)	− 0.143*** (0.023)	5.681*** (0.286)	0.343*** (0.105)	1.272*** (0.160)
Log of spousal earnings	0.052*** (0.008)	0.114*** (0.006)	0.004** (0.002)	− 0.066*** (0.019)	0.006 (0.009)	− 0.112*** (0.016)
Principal component 1	10.063* (5.180)	5.781 (4.470)	7.357*** (2.062)	14.268 (11.906)	− 3.571 (5.140)	13.149 (9.887)
Principal component 2	− 0.036 (5.016)	− 0.947 (4.359)	− 10.224*** (2.013)	11.627 (12.343)	− 10.172** (4.738)	19.659* (10.441)
Principal component 3	2.483 (4.992)	− 4.258 (4.371)	− 3.074 (2.015)	− 27.561** (11.134)	− 3.151 (4.955)	− 1.713 (9.067)
Principal component 4	7.417 (5.036)	− 0.868 (4.370)	− 0.700 (2.015)	− 7.132 (12.110)	− 5.927 (4.983)	− 7.497 (9.562)
Principal component 5	− 0.428 (5.185)	− 5.055 (4.459)	− 16.090*** (2.056)	2.720 (11.845)	1.062 (5.127)	8.974 (9.827)
Principal component 6	− 4.115 (4.854)	− 3.852 (4.255)	− 2.116 (1.964)	− 4.798 (10.798)	− 4.537 (4.835)	6.657 (8.900)
Principal component 7	4.149 (4.970)	3.721 (4.346)	0.691 (2.003)	2.286 (11.320)	4.572 (4.934)	− 9.497 (9.191)
Principal component 8	1.192 (4.852)	− 0.608 (4.258)	3.242* (1.963)	− 1.948 (10.828)	0.561 (4.834)	− 0.605 (8.851)
Principal component 9	2.788 (4.905)	5.171 (4.280)	− 1.998 (1.973)	− 1.546 (11.005)	− 4.238 (4.891)	13.435 (8.963)
Principal component 10	− 7.417 (4.942)	− 9.594** (4.310)	2.655 (1.984)	10.209 (11.063)	− 2.567 (4.879)	19.426** (9.122)
Constant	15.169*** (0.650)	18.160*** (0.455)	7.885*** (0.135)	− 17.995*** (1.767)	− 1.583** (0.747)	− 9.385*** (1.231)

Full regression results for Table 2 (Panel D)

Standard errors in parentheses

****p *< 0.01, ***p *< 0.05, **p *< 0.10

**Table 9:** The relationship between the polygenic risk score (PRS) for ADHD and labor market outcomes (random effects panel regressions)

	(1)	(2)	(3)	(4)	(5)	(6)
	Employed	Log of earnings	Log of household wealth	Receiving social security disability benefits	Receiving unemployment or worker compensation	Receiving other governmental transfers
*Panel E: females and males aged 50–55 (N*_*individuals*_ * = 6279, N*_*individual-wave*_ * = 12,907)*						
PRS for ADHD	− 0.090 (0.059)	− 0.157*** (0.047)	− 0.139*** (0.022)	0.049 (0.153)	0.063 (0.064)	0.305*** (0.107)
Age	− 0.133*** (0.025)	− 0.154*** (0.017)	0.056*** (0.005)	0.262*** (0.073)	0.002 (0.031)	0.079 (0.049)
Female	− 1.030*** (0.128)	− 0.807*** (0.096)	0.265*** (0.044)	− 1.071*** (0.317)	− 0.948*** (0.132)	− 1.559*** (0.225)
With a partner	− 0.704*** (0.167)	− 0.957*** (0.126)	1.002*** (0.048)	− 1.584*** (0.385)	− 0.274 (0.183)	− 1.269*** (0.255)
Number of living children	− 0.021 (0.032)	− 0.015 (0.026)	− 0.083*** (0.011)	0.106 (0.078)	0.053 (0.035)	0.236*** (0.053)
Self-reported health (excellent)	Reference	Reference	Reference	Reference	Reference	Reference
Self-reported health (very good)	0.002 (0.127)	0.064 (0.087)	− 0.099*** (0.028)	− 0.455 (0.619)	0.271* (0.154)	0.598** (0.290)
Self-reported health (good)	− 0.177 (0.142)	− 0.114 (0.100)	− 0.240*** (0.034)	0.726 (0.575)	0.457*** (0.165)	1.020*** (0.300)
Self-reported health (fair)	− 0.861*** (0.180)	− 0.651*** (0.137)	− 0.385*** (0.046)	2.178*** (0.590)	0.352 (0.219)	1.724*** (0.340)
Self-reported health (poor)	− 2.922*** (0.279)	− 2.320*** (0.217)	− 0.803*** (0.073)	3.244*** (0.626)	0.742** (0.308)	2.215*** (0.424)
Health limits work	0.069*** (0.007)	0.078*** (0.005)	0.032*** (0.002)	− 0.095*** (0.019)	− 0.020*** (0.007)	− 0.060*** (0.012)
Tenure in current occupation	− 2.977*** (0.140)	− 2.019*** (0.105)	− 0.227*** (0.035)	6.691*** (0.520)	0.397** (0.157)	1.690*** (0.220)
Log of spousal earnings	0.044*** (0.011)	0.088*** (0.008)	0.008*** (0.003)	− 0.069** (0.032)	0.002 (0.013)	− 0.125*** (0.022)
Principal component 1	9.975 (6.741)	5.754 (5.196)	8.287*** (2.388)	5.470 (17.667)	− 8.145 (7.367)	20.898* (12.527)
Principal component 2	5.823 (6.340)	− 4.013 (5.032)	− 10.992*** (2.313)	36.536* (19.755)	− 16.741*** (6.371)	19.063 (12.666)
Principal component 3	1.228 (6.332)	− 5.490 (5.083)	− 2.498 (2.337)	− 17.437 (16.239)	− 5.458 (6.790)	7.116 (11.119)
Principal component 4	− 0.782 (6.529)	− 0.903 (5.141)	− 1.155 (2.364)	− 25.878 (18.539)	− 5.274 (6.980)	− 6.012 (11.917)
Principal component 5	− 7.386 (6.778)	− 3.702 (5.194)	− 15.631*** (2.385)	9.389 (17.812)	4.889 (7.380)	14.785 (12.461)
Principal component 6	− 2.073 (6.195)	− 2.903 (4.935)	− 3.501 (2.272)	8.650 (15.627)	− 1.037 (6.650)	3.611 (10.869)
Principal component 7	− 0.263 (6.385)	1.448 (5.089)	− 1.051 (2.336)	− 3.400 (16.721)	2.006 (6.851)	− 6.162 (11.344)
Principal component 8	− 3.637 (6.209)	2.070 (4.949)	4.027* (2.272)	− 20.326 (16.023)	− 1.847 (6.671)	0.720 (10.878)
Principal component 9	4.687 (6.256)	6.370 (4.989)	− 1.655 (2.293)	− 6.778 (16.253)	0.381 (6.745)	3.827 (11.057)
Principal component 10	− 7.066 (6.309)	− 10.118** (5.048)	2.230 (2.316)	24.516 (16.473)	2.390 (6.822)	28.212** (11.375)
Constant	11.038*** (1.369)	16.193*** (0.920)	7.678*** (0.276)	− 23.004*** (4.105)	− 4.062** (1.666)	− 9.862*** (2.626)

Full regression results for Table 2 (Panel E)

Standard errors in parentheses

****p *< 0.01, ***p *< 0.05, **p *< 0.10

**Table 10:** The relationship between the polygenic risk score (PRS) for ADHD and labor market outcomes (random effects panel regressions)

	(1)	(2)	(3)	(4)	(5)	(6)
	Employed	Log of earnings	Log of household wealth	Receiving social security disability benefits	Receiving unemployment or worker compensation	Receiving other governmental transfers
*Panel A: females and males (N*_*individuals*_ * = 9033, N*_*individual-wave*_ * = 43,485)*						
PRS for ADHD	− 0.072* (0.037)	− 0.118*** (0.037)	− 0.076*** (0.016)	0.137* (0.082)	0.027 (0.038)	0.204*** (0.067)
Years of education	0.128*** (0.015)	0.196*** (0.015)	0.210*** (0.007)	− 0.212*** (0.033)	− 0.155*** (0.016)	− 0.139*** (0.027)
Age	− 0.297*** (0.006)	− 0.274*** (0.005)	0.046*** (0.001)	0.157*** (0.014)	− 0.075*** (0.007)	0.111*** (0.011)
Female	− 0.974*** (0.077)	− 0.685*** (0.075)	0.186*** (0.033)	− 0.886*** (0.169)	− 0.882*** (0.078)	− 1.733*** (0.142)
With a partner	− 0.883*** (0.077)	− 1.326*** (0.075)	0.812*** (0.023)	− 0.765*** (0.160)	− 0.496*** (0.096)	− 1.105*** (0.132)
Number of living children	0.010 (0.018)	0.049*** (0.018)	− 0.039*** (0.007)	− 0.003 (0.038)	0.022 (0.020)	0.156*** (0.031)
Self-reported health (excellent)	Reference	Reference	Reference	Reference	Reference	Reference
Self-reported health (very good)	− 0.005 (0.060)	0.032 (0.056)	− 0.011 (0.015)	0.180 (0.286)	0.137 (0.089)	0.329** (0.150)
Self-reported health (good)	0.016 (0.069)	0.052 (0.065)	− 0.078*** (0.018)	1.046*** (0.282)	0.259*** (0.096)	0.796*** (0.159)
Self-reported health (fair)	− 0.332*** (0.089)	− 0.142* (0.086)	− 0.183*** (0.024)	2.000*** (0.289)	0.383*** (0.119)	1.300*** (0.181)
Self-reported health (poor)	− 1.634*** (0.141)	− 1.038*** (0.132)	− 0.396*** (0.037)	2.420*** (0.306)	0.459*** (0.171)	1.646*** (0.226)
Health limits work	0.011*** (0.004)	0.054*** (0.004)	0.028*** (0.001)	− 0.076*** (0.009)	− 0.017*** (0.004)	− 0.056*** (0.007)
Tenure in current occupation	− 2.313*** (0.062)	− 2.074*** (0.060)	− 0.149*** (0.016)	4.842*** (0.184)	0.187** (0.081)	0.928*** (0.110)
Log of spousal earnings	0.068*** (0.005)	0.136*** (0.005)	0.001 (0.001)	− 0.069*** (0.014)	0.020*** (0.007)	− 0.089*** (0.012)
Principal component 1	7.279* (4.169)	2.760 (4.072)	0.479 (1.821)	13.589 (9.567)	− 2.077 (4.243)	11.646 (7.767)
Principal component 2	2.940 (3.926)	1.665 (3.945)	− 8.891*** (1.769)	13.454 (9.504)	− 14.864*** (3.833)	29.753*** (8.235)
Principal component 3	− 0.772 (3.962)	0.716 (3.957)	− 1.131 (1.768)	− 13.488 (8.622)	− 2.686 (4.106)	− 8.277 (7.113)
Principal component 4	8.964** (3.937)	0.586 (3.943)	0.193 (1.764)	− 0.281 (9.326)	− 6.677 (4.123)	− 6.064 (7.253)
Principal component 5	− 6.327 (4.175)	− 2.212 (4.067)	− 9.715*** (1.820)	0.096 (9.628)	− 4.237 (4.248)	9.813 (7.777)
Principal component 6	− 6.012 (3.860)	− 4.511 (3.861)	− 0.471 (1.728)	− 3.511 (8.450)	− 1.377 (4.005)	2.021 (6.961)
Principal component 7	− 3.111 (3.915)	4.538 (3.923)	− 0.117 (1.755)	− 1.049 (8.642)	2.177 (4.041)	− 5.687 (7.104)
Principal component 8	− 2.052 (3.858)	− 0.389 (3.862)	1.796 (1.727)	− 1.393 (8.356)	− 2.568 (4.006)	− 1.814 (6.903)
Principal component 9	− 0.909 (3.875)	4.435 (3.869)	− 1.351 (1.731)	0.590 (8.474)	− 3.264 (4.016)	12.376* (6.965)
Principal component 10	− 4.652 (3.920)	− 8.699** (3.926)	3.434* (1.755)	6.348 (8.577)	− 3.387 (4.072)	14.451** (7.137)
Constant	18.620*** (0.415)	20.199*** (0.350)	5.368*** (0.121)	− 14.248*** (1.025)	2.895*** (0.474)	− 10.666*** (0.751)

Full regression results for Table 3 (Panel A)

Standard errors in parentheses

****p *< 0.01, ***p *< 0.05, **p *< 0.10

**Table 11:** The relationship between the polygenic risk score (PRS) for ADHD and labor market outcomes (random effects panel regressions)

	(1)	(2)	(3)	(4)	(5)	(6)
	Employed	Log of earnings	Log of household wealth	Receiving social security disability benefits	Receiving unemployment or worker compensation	Receiving other governmental transfers
*Panel B: females (N*_*individuals*_ * = 4921, N*_*individual-wave*_ * = 24,428)*						
PRS for ADHD	− 0.057 (0.049)	− 0.089* (0.049)	− 0.097*** (0.023)	0.212* (0.112)	0.056 (0.055)	0.169* (0.094)
Years of education	0.123*** (0.022)	0.207*** (0.022)	0.232*** (0.010)	− 0.228*** (0.050)	− 0.129*** (0.025)	− 0.235*** (0.044)
Age	− 0.273*** (0.007)	− 0.260*** (0.006)	0.044*** (0.002)	0.158*** (0.019)	− 0.082*** (0.010)	0.023 (0.014)
Female						
With a partner	− 1.213*** (0.094)	− 1.623*** (0.091)	0.892*** (0.030)	− 0.886*** (0.201)	− 0.623*** (0.129)	− 1.888*** (0.179)
Number of living children	− 0.025 (0.024)	0.012 (0.024)	− 0.016* (0.009)	0.041 (0.051)	0.028 (0.028)	0.146*** (0.042)
Self-reported health (excellent)	Reference	Reference	Reference	Reference	Reference	Reference
Self-reported health (very good)	0.069 (0.076)	0.094 (0.072)	− 0.044** (0.021)	0.484 (0.397)	0.248* (0.133)	0.600*** (0.230)
Self-reported health (good)	0.142 (0.089)	0.150* (0.085)	− 0.121*** (0.025)	1.463*** (0.399)	0.344** (0.144)	1.053*** (0.244)
Self-reported health (fair)	− 0.223* (0.116)	− 0.101 (0.112)	− 0.220*** (0.033)	2.297*** (0.409)	0.677*** (0.175)	1.688*** (0.268)
Self-reported health (poor)	− 1.459*** (0.186)	− 0.771*** (0.171)	− 0.404*** (0.051)	2.581*** (0.432)	0.324 (0.270)	2.254*** (0.318)
Health limits work	0.035*** (0.005)	0.088*** (0.005)	0.028*** (0.002)	− 0.060*** (0.013)	− 0.001 (0.006)	− 0.057*** (0.011)
Tenure in current occupation	− 2.291*** (0.082)	− 1.908*** (0.076)	− 0.169*** (0.022)	4.497*** (0.244)	0.147 (0.119)	1.006*** (0.152)
Log of spousal earnings	0.064*** (0.006)	0.131*** (0.006)	0.000 (0.002)	− 0.077*** (0.018)	0.009 (0.011)	− 0.119*** (0.018)
Principal component 1	5.005 (5.634)	1.971 (5.628)	− 1.047 (2.653)	15.500 (12.934)	2.785 (6.100)	15.659 (11.108)
Principal component 2	8.310 (5.278)	− 0.471 (5.342)	− 13.861*** (2.521)	23.979* (13.646)	− 22.046*** (5.420)	23.607** (11.900)
Principal component 3	− 0.727 (5.256)	2.496 (5.294)	− 3.895 (2.490)	− 9.997 (11.611)	− 6.443 (5.907)	− 1.805 (9.833)
Principal component 4	4.629 (5.280)	0.565 (5.317)	− 1.296 (2.505)	6.062 (12.940)	− 5.392 (5.979)	− 7.075 (10.645)
Principal component 5	0.695 (5.670)	− 0.807 (5.665)	− 11.398*** (2.668)	− 10.436 (13.026)	− 8.655 (6.174)	− 1.347 (11.144)
Principal component 6	− 6.179 (5.176)	− 9.246* (5.207)	0.084 (2.454)	− 7.605 (11.730)	− 4.437 (5.787)	5.520 (9.970)
Principal component 7	− 1.233 (5.258)	6.911 (5.296)	− 2.652 (2.492)	0.190 (11.883)	− 1.016 (5.911)	− 8.096 (10.212)
Principal component 8	1.065 (5.118)	4.724 (5.153)	− 0.164 (2.425)	− 4.578 (11.512)	1.034 (5.766)	− 9.959 (9.803)
Principal component 9	− 2.101 (5.222)	− 2.981 (5.248)	− 3.836 (2.471)	12.223 (11.848)	− 9.092 (5.926)	12.439 (9.957)
Principal component 10	− 7.160 (5.234)	− 13.316** (5.279)	6.771*** (2.483)	0.660 (11.783)	− 7.258 (5.826)	9.486 (10.036)
Constant	16.241*** (0.528)	18.315*** (0.468)	5.302*** (0.175)	− 15.522*** (1.440)	1.899*** (0.709)	− 4.042*** (1.052)

Full regression results for Table 3 (Panel B)

Standard errors in parentheses

****p *< 0.01, ***p *< 0.05, **p *< 0.10

**Table 12:** The relationship between the polygenic risk score (PRS) for ADHD and labor market outcomes (random effects panel regressions)

	(1)	(2)	(3)	(4)	(5)	(6)
	Employed	Log of earnings	Log of household wealth	Receiving social security disability benefits	Receiving unemployment or worker compensation	Receiving other governmental transfers
*Panel C: males (N*_*individuals*_ * = 4112, N*_*individual-wave*_ * = 19,057)*						
PRS for ADHD	− 0.082 (0.055)	− 0.146*** (0.054)	− 0.052** (0.023)	0.035 (0.121)	0.010 (0.053)	0.256** (0.104)
Years of education	0.114*** (0.021)	0.162*** (0.021)	0.191*** (0.009)	− 0.203*** (0.044)	− 0.177*** (0.021)	− 0.023 (0.039)
Age	− 0.346*** (0.010)	− 0.299*** (0.007)	0.050*** (0.002)	0.152*** (0.021)	− 0.070*** (0.010)	0.194*** (0.017)
Female						
With a partner	0.001 (0.132)	− 0.561*** (0.129)	0.657*** (0.036)	− 0.448 (0.274)	− 0.284** (0.145)	0.281 (0.242)
Number of living children	0.068** (0.028)	0.112*** (0.027)	− 0.062*** (0.009)	− 0.070 (0.060)	0.022 (0.028)	0.074 (0.050)
Self-reported health (excellent)	Reference	Reference	Reference	Reference	Reference	Reference
Self-reported health (very good)	− 0.128 (0.099)	− 0.047 (0.087)	0.028 (0.021)	− 0.150 (0.417)	0.039 (0.121)	0.188 (0.214)
Self-reported health (good)	− 0.181* (0.109)	− 0.081 (0.100)	− 0.025 (0.025)	0.599 (0.403)	0.186 (0.128)	0.660*** (0.228)
Self-reported health (fair)	− 0.510*** (0.138)	− 0.202 (0.132)	− 0.139*** (0.033)	1.703*** (0.413)	0.122 (0.162)	1.024*** (0.270)
Self-reported health (poor)	− 1.963*** (0.217)	− 1.445*** (0.206)	− 0.387*** (0.051)	2.289*** (0.437)	0.531** (0.225)	0.959*** (0.348)
Health limits work	− 0.018*** (0.005)	0.016*** (0.005)	0.029*** (0.002)	− 0.093*** (0.012)	− 0.029*** (0.005)	− 0.057*** (0.010)
Tenure in current occupation	− 2.359*** (0.095)	− 2.304*** (0.094)	− 0.121*** (0.023)	5.285*** (0.291)	0.229** (0.111)	0.830*** (0.169)
Log of spousal earnings	0.077*** (0.008)	0.147*** (0.008)	0.002 (0.002)	− 0.058*** (0.021)	0.030*** (0.010)	− 0.077*** (0.017)
Principal component 1	11.482* (6.100)	3.588 (5.768)	2.513 (2.446)	11.338 (14.700)	− 6.369 (5.915)	7.482 (11.788)
Principal component 2	− 6.579 (5.743)	1.690 (5.710)	− 3.164 (2.431)	− 0.470 (13.179)	− 8.221 (5.406)	26.654** (12.191)
Principal component 3	2.013 (5.879)	0.775 (5.812)	1.854 (2.464)	− 19.164 (13.057)	1.231 (5.702)	− 12.885 (11.199)
Principal component 4	12.708** (5.766)	− 0.574 (5.741)	2.660 (2.437)	− 7.119 (13.651)	− 7.578 (5.696)	− 6.642 (11.082)
Principal component 5	− 16.775*** (6.082)	− 5.106 (5.713)	− 7.756*** (2.427)	10.323 (14.833)	− 0.642 (5.866)	14.040 (11.710)
Principal component 6	− 5.461 (5.645)	1.456 (5.617)	− 1.044 (2.385)	0.060 (12.281)	1.843 (5.538)	− 0.254 (10.714)
Principal component 7	− 6.017 (5.712)	1.778 (5.693)	3.015 (2.419)	− 2.100 (12.760)	5.493 (5.526)	− 3.859 (10.913)
Principal component 8	− 6.327 (5.720)	− 6.448 (5.687)	4.117* (2.416)	2.450 (12.260)	− 5.800 (5.555)	5.724 (10.782)
Principal component 9	− 1.130 (5.641)	10.840* (5.597)	1.748 (2.376)	− 14.084 (12.300)	1.451 (5.462)	10.828 (10.699)
Principal component 10	− 0.638 (5.773)	− 1.925 (5.733)	− 0.436 (2.433)	12.223 (12.626)	1.769 (5.693)	17.525 (11.033)
Constant	21.518*** (0.667)	22.148*** (0.516)	5.528*** (0.159)	− 13.709*** (1.477)	3.003*** (0.632)	− 20.310*** (1.160)

Full regression results for Table 3 (Panel C)

Standard errors in parentheses

****p *< 0.01, ***p *< 0.05, **p *< 0.10

**Table 13:** The relationship between the polygenic risk score (PRS) for ADHD and labor market outcomes (random effects panel regressions)

	(1)	(2)	(3)	(4)	(5)	(6)
	Employed	Log of earnings	Log of household wealth	Receiving social security disability benefits	Receiving unemployment or worker compensation	Receiving other governmental transfers
*Panel D: females and males aged 50–59 (N*_*individuals*_ * = 8056, N*_*individual-wave*_ * = 25,556)*						
PRS for ADHD	− 0.052 (0.047)	− 0.111*** (0.040)	− 0.069*** (0.018)	0.126 (0.106)	0.048 (0.046)	0.279*** (0.088)
Years of education	0.121*** (0.020)	0.197*** (0.017)	0.207*** (0.008)	− 0.179*** (0.044)	− 0.178*** (0.020)	− 0.135*** (0.036)
Age	− 0.200*** (0.011)	− 0.187*** (0.008)	0.052*** (0.002)	0.175*** (0.029)	− 0.038*** (0.013)	0.074*** (0.022)
Female	− 1.086*** (0.098)	− 0.739*** (0.082)	0.227*** (0.037)	− 1.132*** (0.219)	− 0.898*** (0.094)	− 1.779*** (0.199)
With a partner	− 0.869*** (0.115)	− 1.196*** (0.094)	0.932*** (0.032)	− 1.021*** (0.238)	− 0.419*** (0.123)	− 1.149*** (0.187)
Number of living children	0.016 (0.024)	0.036* (0.021)	− 0.048*** (0.008)	− 0.036 (0.052)	0.013 (0.025)	0.179*** (0.041)
Self-reported health (excellent)	Reference	Reference	Reference	Reference	Reference	Reference
Self-reported health (very good)	− 0.068 (0.088)	0.016 (0.065)	− 0.031 (0.020)	0.026 (0.395)	0.218** (0.109)	0.281 (0.206)
Self-reported health (good)	− 0.056 (0.100)	0.043 (0.076)	− 0.110*** (0.023)	0.917** (0.381)	0.277** (0.118)	0.792*** (0.220)
Self-reported health (fair)	− 0.609*** (0.126)	− 0.360*** (0.102)	− 0.268*** (0.032)	1.995*** (0.393)	0.283* (0.150)	1.291*** (0.251)
Self-reported health (poor)	− 2.160*** (0.191)	− 1.537*** (0.158)	− 0.520*** (0.049)	2.634*** (0.413)	0.490** (0.211)	1.653*** (0.302)
Health limits work	0.036*** (0.005)	0.064*** (0.004)	0.029*** (0.002)	− 0.084*** (0.012)	− 0.019*** (0.005)	− 0.061*** (0.010)
Tenure in current occupation	− 2.803*** (0.093)	− 1.994*** (0.076)	− 0.125*** (0.023)	5.663*** (0.289)	0.316*** (0.105)	1.252*** (0.161)
Log of spousal earnings	0.050*** (0.008)	0.110*** (0.006)	0.002 (0.002)	− 0.060*** (0.020)	0.011 (0.009)	− 0.108*** (0.017)
Principal component 1	7.237 (5.210)	1.055 (4.457)	2.258 (2.000)	18.108 (12.055)	0.650 (5.138)	16.167 (10.019)
Principal component 2	1.207 (5.023)	0.777 (4.330)	− 8.215*** (1.945)	8.773 (12.409)	− 11.887** (4.714)	17.980* (10.474)
Principal component 3	3.261 (5.001)	− 3.027 (4.341)	− 1.709 (1.946)	− 27.323** (11.226)	− 4.458 (4.930)	− 2.140 (9.117)
Principal component 4	7.698 (5.051)	− 0.191 (4.340)	0.034 (1.946)	− 7.997 (12.275)	− 6.835 (4.996)	− 8.148 (9.690)
Principal component 5	1.972 (5.213)	− 0.897 (4.441)	− 11.380*** (1.993)	− 1.148 (11.998)	− 2.586 (5.127)	6.244 (9.924)
Principal component 6	− 4.054 (4.861)	− 3.832 (4.224)	− 1.948 (1.896)	− 5.760 (10.899)	− 4.433 (4.813)	6.875 (8.956)
Principal component 7	3.826 (4.977)	3.353 (4.315)	0.281 (1.935)	2.471 (11.413)	4.620 (4.904)	− 9.738 (9.255)
Principal component 8	0.795 (4.859)	− 1.350 (4.228)	2.433 (1.896)	− 0.399 (10.914)	1.482 (4.810)	0.207 (8.907)
Principal component 9	2.820 (4.912)	5.103 (4.250)	− 2.011 (1.905)	− 2.909 (11.100)	− 3.854 (4.858)	13.469 (9.017)
Principal component 10	− 7.155 (4.951)	− 9.003** (4.279)	3.184* (1.916)	10.193 (11.165)	− 3.177 (4.860)	19.163** (9.202)
Constant	13.458*** (0.698)	15.374*** (0.514)	4.989*** (0.170)	− 15.458*** (1.850)	1.012 (0.798)	− 7.554*** (1.311)

Full regression results for Table 3 (Panel D)

Standard errors in parentheses

****p *< 0.01, ***p *< 0.05, **p *< 0.10

**Table 14:** The relationship between the polygenic risk score (PRS) for ADHD and labor market outcomes (random effects panel regressions)

	(1)	(2)	(3)	(4)	(5)	(6)
	Employed	Log of earnings	Log of household wealth	Receiving social security disability benefits	Receiving unemployment or worker compensation	Receiving other governmental transfers
*Panel E: females and males aged 50–55 (N*_*individuals*_ * = 6279, N*_*individual-wave*_ * = 12,907)*						
PRS for ADHD	− 0.054 (0.059)	− 0.103** (0.047)	− 0.080*** (0.021)	− 0.020 (0.155)	0.012 (0.064)	0.264** (0.108)
Years of education	0.147*** (0.026)	0.208*** (0.020)	0.213*** (0.009)	− 0.242*** (0.066)	− 0.200*** (0.028)	− 0.182*** (0.045)
Age	− 0.134*** (0.025)	− 0.155*** (0.017)	0.055*** (0.005)	0.260*** (0.074)	0.002 (0.031)	0.078 (0.049)
Female	− 1.016*** (0.128)	− 0.780*** (0.096)	0.291*** (0.042)	− 1.040*** (0.319)	− 0.966*** (0.132)	− 1.584*** (0.228)
With a partner	− 0.676*** (0.168)	− 0.937*** (0.126)	1.017*** (0.047)	− 1.730*** (0.393)	− 0.317* (0.183)	− 1.322*** (0.258)
Number of living children	0.011 (0.033)	0.030 (0.026)	− 0.042*** (0.011)	0.066 (0.079)	0.011 (0.035)	0.205*** (0.054)
Self-reported health (excellent)	Reference	Reference	Reference	Reference	Reference	Reference
Self-reported health (very good)	0.053 (0.128)	0.126 (0.087)	− 0.067** (0.028)	− 0.513 (0.625)	0.202 (0.154)	0.526* (0.292)
Self-reported health (good)	− 0.076 (0.143)	0.016 (0.101)	− 0.171*** (0.033)	0.595 (0.581)	0.308* (0.166)	0.875*** (0.303)
Self-reported health (fair)	− 0.709*** (0.181)	− 0.453*** (0.138)	− 0.276*** (0.046)	1.974*** (0.595)	0.124 (0.220)	1.509*** (0.345)
Self-reported health (poor)	− 2.719*** (0.280)	− 2.053*** (0.217)	− 0.647*** (0.072)	2.972*** (0.631)	0.446 (0.308)	1.957*** (0.428)
Health limits work	0.067*** (0.007)	0.076*** (0.005)	0.029*** (0.002)	− 0.089*** (0.019)	− 0.019*** (0.007)	− 0.056*** (0.012)
Tenure in current occupation	− 2.960*** (0.140)	− 2.000*** (0.105)	− 0.208*** (0.034)	6.683*** (0.529)	0.366** (0.156)	1.681*** (0.222)
Log of spousal earnings	0.040*** (0.011)	0.083*** (0.008)	0.005* (0.003)	− 0.054* (0.032)	0.008 (0.013)	− 0.119*** (0.022)
Principal component 1	6.730 (6.783)	1.067 (5.175)	3.364 (2.306)	9.288 (17.977)	− 3.552 (7.371)	24.839* (12.718)
Principal component 2	7.513 (6.351)	− 2.183 (4.994)	− 8.944*** (2.226)	32.171 (19.801)	− 18.563*** (6.367)	16.370 (12.718)
Principal component 3	1.946 (6.344)	− 4.433 (5.043)	− 1.424 (2.248)	− 17.108 (16.344)	− 6.732 (6.766)	6.950 (11.218)
Principal component 4	− 0.594 (6.556)	0.033 (5.100)	− 0.234 (2.274)	− 27.075 (18.820)	− 6.054 (7.026)	− 6.597 (12.156)
Principal component 5	− 4.903 (6.813)	0.350 (5.167)	− 11.102*** (2.302)	5.630 (18.168)	1.155 (7.393)	11.301 (12.637)
Principal component 6	− 2.081 (6.205)	− 3.248 (4.896)	− 3.792* (2.185)	7.459 (15.744)	− 0.556 (6.628)	3.967 (10.975)
Principal component 7	− 0.405 (6.393)	1.285 (5.048)	− 1.230 (2.247)	− 4.330 (16.856)	1.785 (6.818)	− 6.860 (11.458)
Principal component 8	− 4.209 (6.219)	1.148 (4.911)	2.964 (2.186)	− 17.316 (16.115)	− 0.765 (6.640)	1.878 (10.975)
Principal component 9	4.753 (6.265)	6.461 (4.949)	− 1.389 (2.205)	− 9.696 (16.403)	0.743 (6.702)	3.481 (11.149)
Principal component 10	− 7.025 (6.325)	− 9.571* (5.008)	2.810 (2.228)	26.304 (16.627)	2.225 (6.812)	28.239** (11.510)
Constant	8.959*** (1.409)	13.239*** (0.963)	4.695*** (0.301)	− 19.570*** (4.162)	− 1.130 (1.713)	− 7.243*** (2.705)

Full regression results for Table 3 (Panel E)

Standard errors in parentheses

****p *< 0.01, ***p *< 0.05, **p *< 0.10

**Table 15:** The indirect relationship between the polygenic risk score (PRS) for ADHD and labor market outcomes through educational attainment (reduced set of control variables)

	(1)	(2)	(3)	(4)	(5)	(6)
	Employed	Log of earnings	Log of household wealth	Receiving social security disability benefits	Receiving unemployment or worker compensation	Receiving other governmental transfers
*Panel A: females and males (N*_*individuals*_ * = 9033, N*_*individual-wave*_ * = 43,485)*						
Indirect effect via years of education	− 0.078*** (0.006)	− 0.094*** (0.006)	− 0.077*** (0.004)	0.108*** (0.013)	0.057*** (0.005)	0.092*** (0.009)
Proportion of mediation	32.31%	31.57%	42.30%	17.87%	53.42%	21.40%

Standard errors in parentheses

****p *< 0.01, ***p *< 0.05, **p *< 0.1

**Table 16:** The indirect relationship between the polygenic risk score (PRS) for ADHD and labor market outcomes through educational attainment (including control variables for census region of birth and the interaction between age and census region of birth)

	(1)	(2)	(3)	(4)	(5)	(6)
	Employed	Log of earnings	Log of household wealth	Receiving social security disability benefits	Receiving unemployment or worker compensation	Receiving other governmental transfers
*Panel A: females and males (N*_*individuals*_ * = 9029, N*_*individual-wave*_ * = 43,472)*						
Indirect effect via years of education	− 0.031*** (0.004)	− 0.045*** (0.004)	− 0.049*** (0.003)	0.046*** (0.008)	0.038** (0.004)	0.031*** (0.007)
Proportion of mediation	31.86%	28.31%	40.57%	29.11%	53.25%	13.65%

Standard errors in parentheses

****p *< 0.01, ***p *< 0.05, **p *< 0.10

## Footnotes

1 The Rand HRS data file Version P includes harmonized data from the 1992, 1993, 1994, 1995, 1996, 1998, 2000, 2002, 2004, 2006, 2008, 2010, 2012, and 2014 data collection waves.

2 This procedure decomposes the total effect of the PRS for ADHD on the labor market outcomes into direct and indirect (through years of education) effects and has the advantage of providing unbiased decompositions in non-linear models (such as the logit model for the binary outcomes).

3 Full regression results are available in the “Appendix”.

4 The PRS for ADHD may not only influence educational attainment, but also some of our control variables. To address this issue, we re-estimated the indirect effects in a model with only gender, age, and the principal components as control variables. The results are in line with the main results (see Table 15 in the “Appendix”). In addition, educational attainment in terms of years of education is partly the result of prevailing schooling laws which may have been different across regions and time. To address this issue, we re-estimated the indirect effects in a model with additional control variables for 11 census regions of birth and the interaction between age and census region of birth. The results are in line with the main results (see Table 16 in the “Appendix”).
